# Efficacy Evaluation of Tissue Plasminogen Activator with Anti-Vascular Endothelial Growth Factor Drugs for Submacular Hemorrhage Treatment: A Meta-Analysis

**DOI:** 10.3390/jcm12031035

**Published:** 2023-01-29

**Authors:** Xuejun He, Wenye Cao, Zhiyi Wang, Ningzhi Zhang, Kexin Xu, Lu Yu, Yiqiao Xing, Ning Yang

**Affiliations:** Eye Center, Renmin Hospital of Wuhan University, 238 Jiefang Road, Wuhan 430060, China

**Keywords:** submacular hemorrhage, tissue plasminogen activator, anti-vascular endothelial growth factor, combination treatment, meta-analysis

## Abstract

Submacular hemorrhage (SMH) is the accumulation of blood in the macular area that can severely damage the macular structure and visual function. Recently, the intraocular administration of tissue plasminogen activator (TPA) with anti-vascular endothelial growth factor (anti-VEGF) drugs was reported to have a positive effect on SMH. This meta-analysis aimed to explore the efficacy and safety of the drug combination. We systematically searched the Web of Science, MEDLINE, EMBASE, and Cochrane Library databases and screened relevant full-length literature reports. The quality of the reports was assessed by two independent reviewers. The best-corrected visual acuity (BCVA) and foveal thickness (FT) were considered the main indicators of efficacy. RevMan 5.4 software was used for this meta-analysis. Twelve studies were analyzed, and the results showed that BCVA at 1 month (*p* < 0.001), 3 months (*p* < 0.001), 6 months (*p* < 0.001), and the last follow-up (*p* < 0.001) was improved relative to the preoperative value. The postoperative FT was lower than the preoperative FT (*p* < 0.001). No significant difference in efficacy was observed between subretinal and intravitreal TPA injections (*p* = 0.37). TPA with anti-VEGF drugs is safe for SMH treatment and can significantly improve BCVA and reduce FT.

## 1. Introduction

Submacular hemorrhage (SMH) is characterized by the presence of blood between the retinal pigment epithelium (RPE) and the neurosensory retina in the macular area. It is caused by choroidal and retinal vascular abnormalities [1]. SMH damages photoreceptors in several ways, including iron toxicity, fibrin meshwork contraction, outer retinal shear forces, and reduced nutrient supply, eventually resulting in macular scarring [2]. SMH caused by various ocular diseases such as neovascular age-related macular degeneration (nAMD), polypoid choroidal vasculopathy (PCV), pathological myopia, and retinal aneurysm has a significantly negative impact on the patient’s visual ability, with a poor prognosis [3]. A population-based study in 2014 estimated that the annual incidence of new and large SMH complicated with wet AMD was approximately 25 per million per year, which is detrimental to global eye health [4]. The cellular mechanism of SMH remains to be explored, as recent studies have revealed the importance of oxidative stress and inflammation response in arteriovenous pathologies [5,6]. SMH can result in sudden or progressive vision loss depending on the extent and thickness of the bleeding, and the reception of visual information by photoreceptors can be blocked, with subsequent damage. A retrospective review of eyes with massive SMH confirmed that visual acuity correlated inversely with the thickness rather than the diameter of SMH [7].

Several methods have been used for the treatment of SMH secondary to AMD. Surgical progress mainly focuses on vitrectomy along with multiple procedures such as the removal of choroidal neovascularization (CNV) lesions, macular translocation, RPE patch repair, and gas replacement, followed by intravitreal/subretinal drug injection or SMH drainage [8]. Tissue plasminogen activator (TPA) is a commonly used drug for SMH treatment. It has a molecular weight of 72 kD and a short half-life [8]. The plasminogen is activated by fibrin and transformed into plasmin, which combines with fibrin to dissolve blood clots [9]. Thus, the injection of TPA into the subretinal area would protect the retina by cleaving the fibrin, dissolving the clot, reducing iron toxicity, and improving nutritional supply [8,9]. In the past decades, many studies have shown that TPA injection and pneumatic displacement during surgery are effective in displacing SMH and, consequently, improving postoperative vision. Both intravitreal and subretinal injections of TPA are widely used [10,11,12,13]. Subretinal injection (SRI) of TPA could dissolve the clot and displace SMH, which allows the blood to be evacuated through a small retinotomy. However, this method is risky and can cause complications [14]. The intravitreal injection (IVI) of TPA is considered an alternative therapy because of its efficacy [11]. Because the incidence of complications is lower than that after a single surgery, such as pars plana vitrectomy, it is considered a minimally invasive and valuable approach [15]. TPA and gas can efficiently displace hemorrhage; however, CNV progression after SMH could limit the therapeutic effects of these techniques. In addition, anti-vascular endothelial growth factor (anti-VEGF) drugs are widely used to treat underlying CNV [16]. A few studies have reported that intravitreal anti-VEGF monotherapy could offer moderate visual gain in patients with SMH; however, the co-application of TPA and anti-VEGF is commonly chosen to maintain the treatment effects [16,17,18].

Currently, there is no gold standard treatment principle for SMH, and the order in which TPA and anti-VEGF are used remains unclear, with variable results. Bardak et al. [19] found that SMH displacement was successfully achieved with sequential TPA administration, pneumatic displacement, and anti-VEGF drug therapy in 16 eyes of 16 patients. The postoperative best-corrected visual acuity (BCVA) of all patients was significantly improved at 1, 3, 6, and 12 months. In another study by Avci et al. [20], 30 patients diagnosed with SMH received TPA and anti-VEGF drugs during the surgical procedure. Compared with that at baseline, BCVA significantly improved at 1, 2, 3, and 12 months postoperatively. Furthermore, the group that received additional anti-VEGF therapy showed a significant reduction in the SMH area. The differences in results among these studies could be attributed to differences in the indications for surgery, baseline demographics, and treatment protocols. The aim of this meta-analysis was to evaluate the efficacy and safety of TPA with anti-VEGF therapy for SMH and consolidate the available evidence.

## 2. Materials and Methods

### 2.1. Search Strategy

The Web of Science, MEDLINE, EMBASE, and Cochrane Library databases were systematically searched from inception through 10 June 2022. Keywords included submacular hemorrhage, sub-macular hemorrhage, or SMH; recombinant tissue plasminogen activator, rt-PA, rtPA, r-tPA, or rTPA; and anti-vascular endothelial growth factor, anti-VEGF, ranibizumab, aflibercept, conbercept, or bevacizumab. Only articles published in English were included in the analysis. There were no date restrictions.

### 2.2. Literature Inclusion and Exclusion Criteria

Only clinical trials evaluating the efficacy of combined anti-VEGF and TPA treatments for SMH were included. Case reports, review articles, medical guidelines, abstract-only publications, and conference summaries were excluded. In addition, publications in languages other than English were excluded. Two independent reviewers (X.H. and W.C.) screened the articles by reading the titles, abstracts, keywords, and full texts. Studies that did not report the number of eyes and/or involved a small sample size of fewer than eight eyes were excluded. Moreover, studies were excluded if the group setting or data analysis was inappropriate. Studies based on the sequential application of anti-VEGF drugs and TPA were also excluded. Articles that met the requirements were listed and evaluated to ensure the inclusion of all eligible studies. Disagreements between the researchers were resolved through consultation with a third author (N.Z.). This meta-analysis was performed in accordance with the PRISMA guidelines (registration number CRD42022358037).

### 2.3. Literature Quality Evaluation

Because all the included studies were retrospective in nature, the Newcastle–Ottawa Scale (NOS) [21] was used by the two authors to assess the methodological quality and risk of bias of the studies. NOS is based on a star system for assessing the quality of case-control or cohort studies for seven items categorized into three broad groups: (1) selection (S, four stars), (2) comparability (C, two stars), and (3) exposure or outcome (O, three stars). Thus, the maximum number of stars that a study can receive is nine. Two authors (X.H. and W.C.) independently assessed the quality of the included studies. Disagreements between the researchers were resolved through consultation with a third author (N.Z.).

### 2.4. Outcome Indicator

The primary outcome measures were BCVA and the foveal thickness (FT) after treatment. The secondary outcomes were hemorrhage displacement and postoperative complications.

### 2.5. Data Extraction

Data from the included studies were independently extracted by two authors (X.H., W.C.). Studies with unclear or missing data records were excluded, and disagreements between the researchers were resolved through consultation with a third author (N.Z.). Data were collected and recorded as follows: (1) literature information for the included studies (first author, publication time, country, or region), (2) basic information about the study participants (sample size, age, sex, duration of disease course, baseline BCVA, baseline FT, and intervention measures), and (3) outcomes (BCVA after treatment, FT after treatment, bleeding displacement, and postoperative complications).

### 2.6. Statistical Methods

Data analysis was performed using Review Manager (RevMan 5.4, The Cochrane Collaboration, Oxford, UK) software. Continuous variables were statistically analyzed using the mean difference and are reported as the weighted mean deviation (WMD) with a 95% confidence interval (CI). Forest plots were used to describe and represent the statistical results.

When follow-up data were available at several time points in the study, the data at each follow-up and the final reported data were extracted. A paired-samples test was used to compare data before and after treatment.

The I^2^ statistic was used to evaluate the heterogeneity of the results. I^2^ ≤ 50% indicated low heterogeneity, and a fixed effects model was used. When I^2^ was >50%, the heterogeneity was considered high, and a random effects model was used. Funnel plots were used for the visual assessment of publication bias.

## 3. Results

### 3.1. Search Results

**Figure 1:**
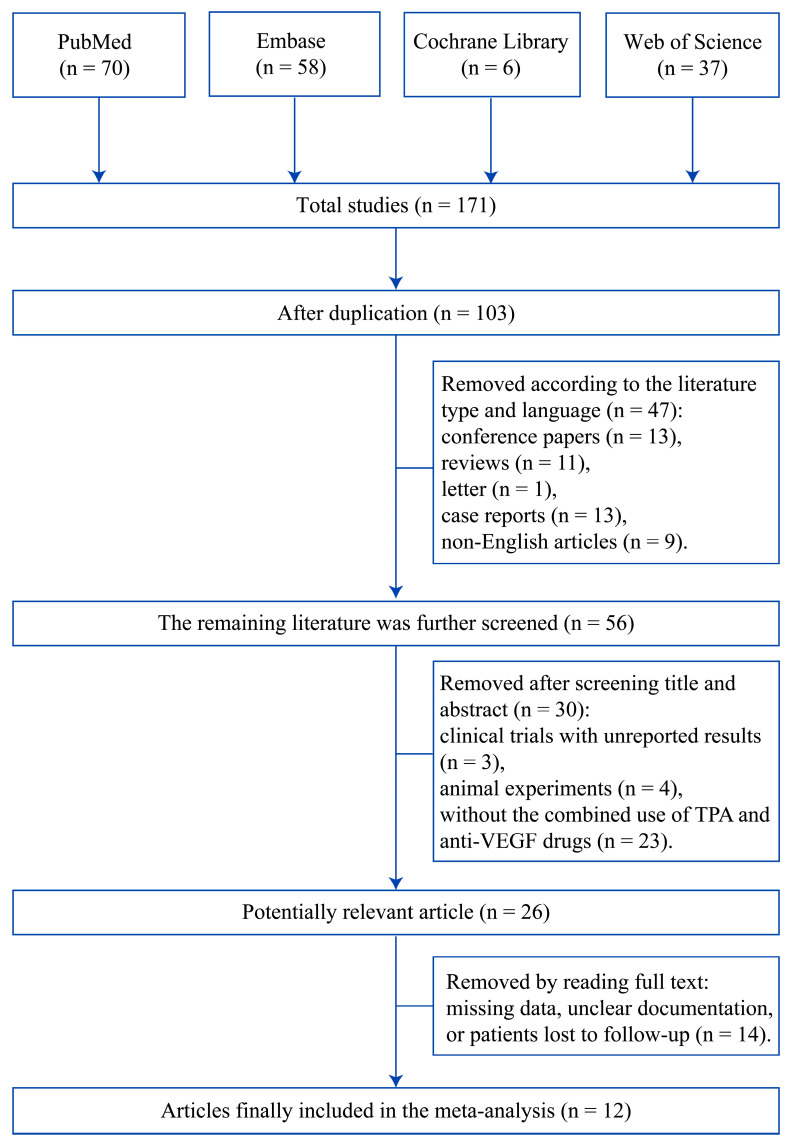
Study selection flowchart.

The study selection flowchart is shown in Figure 1. In total, 171 articles (PubMed (n = 70), Embase (n = 58), Cochrane Library (n = 6), and Web of Science (n = 37)) were identified. No Chinese database was included in this study. Endnote literature management software was used to remove 68 duplicates. Based on the literature type and language, 47 articles were excluded, including 13 conference papers, 11 reviews, one letter, 13 case reports, and nine non-English articles. After reading the title and abstract, 30 articles were eliminated, including three clinical trials with unreported results, four articles on animal experiments, and 23 without the combined use of TPA and anti-VEGF drugs. Subsequently, two independent reviewers screened the full text of 26 possible relevant studies, 14 of which were excluded because of missing data, unclear documentation, or loss of follow-up. Thus, 12 retrospective studies [20,22,23,24,25,26,27,28,29,30,31,32] including a total of 269 eyes from 269 participants were included in this meta-analysis.

### 3.2. Description of the Included Studies

The 12 articles included in this meta-analysis were retrospective studies. Table 1 summarizes the main characteristics of the included studies, including the first author, publication time, sample size, number of eyes, country, etiology of SMH, patient age, disease duration, bleeding area, and NOS star level. Based on the conversion relationship between the bleeding area and the optic disc diameter in the study by Arias et al. [22], the bleeding areas in the studies by Avci et al. [20] and Kitagawa et al. [26] were converted into a representation of the optic disc diameter.

Table 2 presents the treatment modalities, complications, and hemorrhage displacement procedures received by the study participants. Grohmann et al. [31] evaluated the effect of three surgical modalities for SMH treatment and termed them Grohmann G1, G2, or G3 according to the different treatment regimens received by each group. 

The BCVA and FT data before and after SMH treatment are summarized in Table 3 and Table 4, respectively. Based on the conversion scheme of different visual acuity recording methods published by Ferris et al. [33], some of the original values in the studies by Erdogan et al. [30], Kitagawa et al. [26], and Guthoff et al. [24] were converted to logarithms of the minimum angle of resolution units to facilitate subsequent statistical analysis.

### 3.3. Analysis Results

#### 3.3.1. Analysis of BCVA

**Figure 2:**
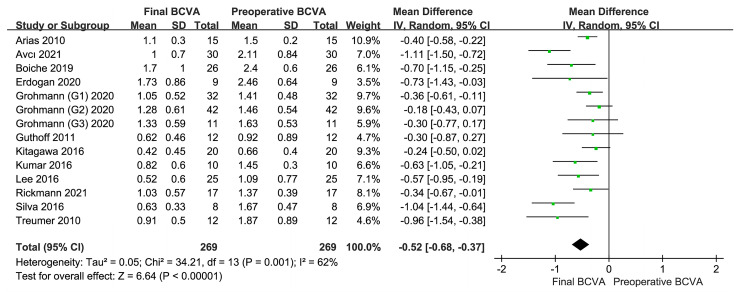
Final BCVA versus preoperative BCVA [20,22,23,24,25,26,27,28,29,30,31,32]. All visual acuity data are expressed in logMAR. The results show that the BCVA of the study participants has significantly improved after combined treatment with TPA and anti-VEGF drugs. BCVA, best-corrected visual acuity; TPA, tissue plasminogen activator; anti-VEGF, anti-vascular endothelial growth factor; logMAR, logarithm of the minimum angle of resolution.

The final BCVA values in the 12 included studies were analyzed, and the comparative results are shown in the first forest plot (Figure 2). The final BCVA significantly improved relative to the initial BCVA (MD = −0.52, 95% CI= (−0.68, −0.37), I^2^ = 62%, *p* < 0.001). Heterogeneity analysis showed that I^2^ (62%) was more than 50%; therefore, a more conservative random effects model was used. The findings suggested that TPA combined with anti-VEGF therapy is effective in improving the final visual acuity of patients with SMH.

**Figure 3:**
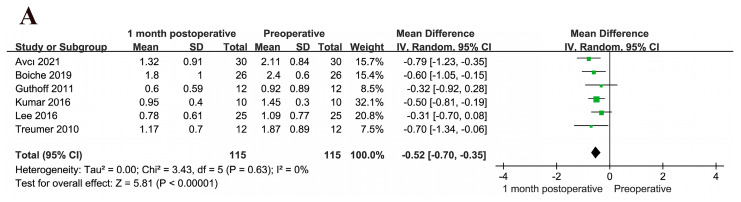

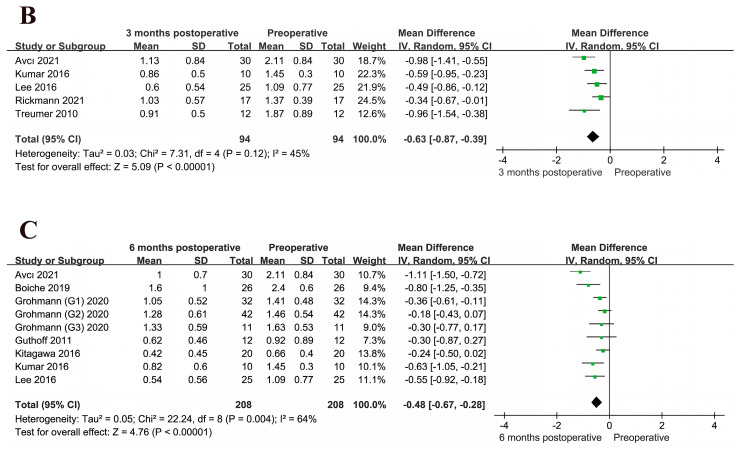
(**A**) BCVA at 1 month postoperatively versus preoperative BCVA [20,23,24,27,28,29]. (**B**) BCVA at 3 months postoperatively versus preoperative BCVA [20,23,27,28,32]. (**C**) BCVA at 6 months postoperatively versus preoperative BCVA [20,24,26,27,28,29,31]. All visual acuity data are expressed in logMAR. BCVA values at the three different follow-up time points are significantly different from the preoperative BCVA. BCVA, best-corrected visual acuity; logMAR, logarithm of the minimum angle of resolution.

Among the included studies, six, five, and seven documented BCVA at 1, 3, and 6 months, respectively. Grohmann et al. [31] studied the influence of different surgical methods (G1, G2, and G3) on the treatment effect. The postoperative BCVA values at 1 (Figure 3A), 3 (Figure 3B), and 6 (Figure 3C) months were better than the preoperative BCVA. The results of the meta-analysis of postoperative BCVA values were as follows: 1 month: MD = −0.52, 95% CI = (−0.70, −0.35), I^2^ = 0%, *p* < 0.001; 3 months: MD = −0.63, 95% CI = (−0.87, −0.39), I^2^ = 45%, *p* < 0.001; and 6 months: MD = −0.48, 95% CI = (−0.67, −0.28), I^2^ = 64%, *p* < 0.001. The differences were statistically significant. The random effects model was used to compare the three groups at the different follow-up time points. The results suggested that TPA with anti-VEGF treatment for SMH significantly improves BCVA and the prognosis in the short and long terms, respectively.

#### 3.3.2. Effect of the Location of TPA Use on BCVA

**Figure 4:**
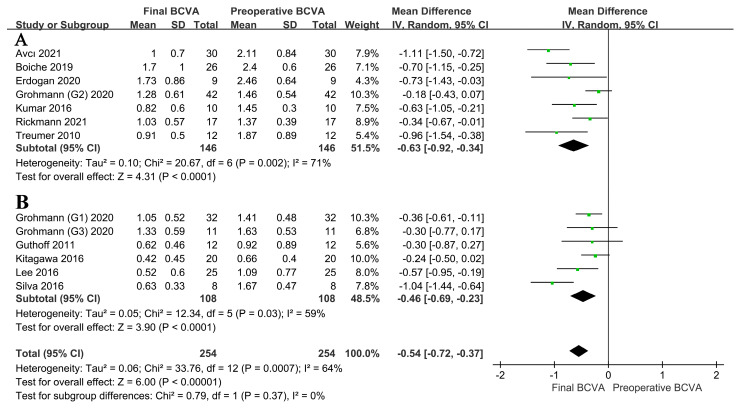
(**A**) BCVA after the SRI [20,23,27,29,30,31,32] of TPA versus (**B**) BCVA after the IVI of TPA [24,25,26,28,31]. Both subgroups show improved postoperative BCVA relative to the preoperative BCVA, with no significant difference between the two subgroups. This indicates that the site of TPA injection does not affect BCVA. BCVA, best-corrected visual acuity; SRI, subretinal injection; TPA, tissue plasminogen activator; IVI, intravitreal injection.

The included studies were divided into two subgroups, SRI and IVI groups, according to the site of TPA injection. The results of this analysis are shown in Figure 4. The study by Arias et al. included both subretinal and intravitreal TPA injections, and the participants were not grouped according to the site of injection; therefore, this subgroup analysis was not included in the meta-analysis. The subgroup analysis showed that the SRI or IVI of TPA could increase BCVA (SRI group: MD = −0.63, 95% CI = (−0.92, −0.34), I^2^ = 71%, *p* < 0.001; IVI group: MD = −0.46, 95% CI = (−0.69, −0.23), I^2^ = 59%, *p* < 0.001; overall: MD = −0.54, 95% CI = (−0.72, −0.37), I^2^ = 64%, *p* = 0.37). A random effects model was used because I^2^ was >50%. Overall, the site of TPA administration did not affect the trend in BCVA improvement (*p* = 0.37 > 0.05 for the difference between groups).

#### 3.3.3. Analysis of Foveal Thickness

**Figure 5:**
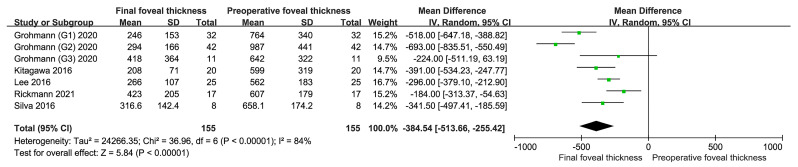
Final foveal thickness versus preoperative foveal thickness [25,26,28,31,32]. The foveal thickness is expressed in microns (μm), and it has decreased after treatment. This indicates that the combination of TPA and anti-VEGF drugs is beneficial for the structural restoration of the fovea. TPA, tissue plasminogen activator; anti-VEGF, anti-vascular endothelial growth factor.

Figure 5 shows the results of comparisons between the final FT and preoperative FT in cases subjected to combined TPA and anti-VEGF treatment for SMH. FT decreased postoperatively, and the difference was statistically significant (MD = −384.54, 95% CI = (−513.66, −255.42), I^2^ = 84%, *p* < 0.001). I^2^ was >50%, and the random effects model was used. The findings indicated that combined treatment with TPA and anti-VEGF drugs can promote a decrease in FT, which is beneficial for the structural recovery of the fovea in patients with SMH.

## 4. Discussion

The accumulation of blood in the macula can cause irreversible damage to photoreceptors within 24 h, and damage to visual function can be particularly serious [34]. The etiology of SMH can generally be divided into two categories: CNV (such as nAMD, PCV, and pathologic myopia) and non-CNV (such as retinal aneurysm, Terson syndrome, and trauma). CNV-associated SMH is more common. The choice of treatment for SMH is closely related to the cause of the hemorrhage. In non-CNV SMH, visual acuity usually improves to varying degrees after the blood is absorbed or cleared [35]. In CNV SMH, retinal neovascularization hinders physiological metabolic processes and acts as a basic factor for hemorrhage. Therefore, a reduction in the damage from hemorrhage in the macula and the inhibition of the persistent impact of neovascularization should be simultaneously achieved.

TPA is widely found in the aqueous humor, vitreous humor, and retina of the eye and plays an important role in eye development [36]. TPA can activate plasminogen into plasmin, which hydrolyzes fibrin and promotes blood clot dissolution and absorption [37]. The retinal expression of VEGF increases under hypoxia and induces neovascularization in the short term [38]. Based on the results of existing clinical trials, this article discusses the influence of the TPA injection site on the efficacy of combination therapy. Dr. Hilel Lewis showed in animals that labeled intravitreally injected TPA was present on the vitreous surface and failed to reach the neural retina or subretinal clots. This indicated that TPA does not diffuse through intact ILM in animals, and there is no scientific basis for the pure IVI of TPA in the treatment of SMH without vitreous hemorrhage, which may be caused by the rupture of the overlying retina [14]. This prompts doctors to select therapies according to the condition in clinical practice. Although we analyzed and concluded that the site of TPA administration does not affect the trend in BCVA improvement, rational selection of the TPA injection site according to the presence or absence of concomitant vitreous hemorrhage may provide greater benefits to patients. Anti-VEGF drugs can effectively inhibit neovascularization and reduce the associated damage to the eye [39]. A large number of studies have shown that the intraocular injection of TPA and anti-VEGF drugs can effectively and safely treat subretinal hemorrhage of different etiologies. However, the efficacy of combining the two drugs for the treatment of CNV SMH has not been systematically analyzed.

This meta-analysis included 269 eyes of 269 participants from 12 articles. Only a few of the reported complication rates in the included studies exceeded 30%, with most ranging from 2.4% to 20%; this indicates that TPA combined with anti-VEGF drugs is relatively safe. BCVA and FT were used as the main indicators of the efficacy of TPA with anti-VEGF drugs for the treatment of SMH. The results showed that TPA with anti-VEGF drug therapy significantly improved the BCVA of patients. At 1, 3, and 6 months after treatment, BCVA improved relative to that before treatment. In addition, five of these studies indicated that the combination of the two drugs reduced FT and promoted the structural recovery of the fovea. Furthermore, no significant differences in BCVA improvement were observed between subretinal and intravitreal TPA injections; this finding was not consistent with those of some other studies. For example, Wilkins et al. [40] and Ohayon et al. [41] found that the SRI of TPA can effectively promote the recovery of vision and facilitate the displacement and absorption of blood. In contrast, Tranos et al. [42] and Bell et al. [43] found that the IVI of TPA is beneficial for reducing the incidence of complications and has the same effect as SRI, consistent with our results. Notably, efficacy was limited by underlying diseases regardless of the type of treatment. Therefore, more research is needed to evaluate the efficacy of TPA and anti-VEGF therapy for SMH complicated by multiple eye diseases besides AMD.

This meta-analysis had some limitations. Several outcomes showed high heterogeneity in this research, probably because of the study population, duration of disease, and measurement methods. Random effects models were used for each of the primary outcomes to make the conclusions more reasonable, and subgroup analyses were performed according to the site of TPA injection. The 12 included articles were all retrospective studies; therefore, randomized controlled studies were lacking. Moreover, after the onset of SMH, patients are expected to receive the best individualized treatment; thus, setting a control group would be morally difficult. Therefore, the NOS scores of the included studies were generally five or seven stars (Table 1). After receiving the combined TPA and anti-VEGF treatment, some patients received additional anti-VEGF treatment, which was not included in this meta-analysis. During SMH treatment, in addition to TPA and anti-VEGF combination therapy, patients may have undergone other treatments such as surgery, gas replacement, and follow-up care, for a full recovery. These treatments are affected by various factors, such as the surgeon’s proficiency, medical conditions, and patient cooperation, and these factors may have introduced bias in the results of the clinical studies and secondary analyses. In addition, the sample size of some studies was slightly small, and multicenter, large-sample randomized controlled trials are needed to overcome the limitations of this study.

With the in-depth study of molecular mechanisms and the application of various experimental techniques, the treatment of SMH will predictably show standardization and precision in the future. Among vascular abnormality-related diseases, cerebral cavernous malformations have gained a lot of attention in recent years, and some studies have more deeply explored the dysregulated pathways such as oxidative stress and inflammation response in the pathogenesis by applying next-generation sequencing technology [5,6]. In ophthalmology, causative genes for abnormal angiogenic pathways are also being investigated [44]. These pioneering results based on transcriptome analysis point to genes that may be involved in pathogenesis and serve as potential therapeutic targets to diversify the diagnosis and treatment of diseases. Therefore, exploring and targeting pathogenesis at the cellular level may also become an important and insightful component of future SMH therapy.

## 5. Conclusions

In summary, this meta-analysis suggests that TPA with anti-VEGF drugs for SMH treatment is safe and can significantly improve BCVA and reduce FT, with no statistically significant difference in the treatment effect between subretinal and intravitreal TPA injections. Further studies should assess the optimal therapeutic doses of various anti-VEGF drugs and focus on monitoring complications.

## Figures and Tables

**Table 1:** Characteristics of the included studies.

Author	Publication Time (Year)	Patient No.			Eye No.	Country	Etiology	Age (Years)		Duration (Days)		Hemorrhage Area (DD)		NOS
		Male	Female	Total				Mean	SD	Mean	SD	Mean	SD	
Arias [22]	2010	5	10	15	15	Spain	AMD	79.6	8.6	≤5		9.8	2.9	☆☆☆☆☆
Avcı [20]	2021	14	16	30	30	Turkey	AMD	73.33	8.23	13.7	8.05	22.7	15.9	☆☆☆☆☆
Boiche [29]	2019	9	17	26	26	French	AMD	78	8	7	4.8	4.2	1.7	☆☆☆☆☆
Erdogan [30]	2020	4	5	9	9	Turkey	AMD	72.2	10.2	15.3	12.8	-	-	☆☆☆☆☆
Grohmann (G1) [31]	2020	-	-	32	32	Germany	AMD	85.36	-	9.1	4.6	4.4	1.8	☆☆☆☆☆☆☆
Grohmann (G2) [31]	2020	-	-	42	42	Germany	AMD	85.36	-	9.1	4.6	4.14	1.3	☆☆☆☆☆☆☆
Grohmann (G3) [31]	2020	-	-	11	11	Germany	AMD	85.36	-	9.1	4.6	4.68	2.8	☆☆☆☆☆☆☆
Guthoff [24]	2011	5	7	12	12	Germany	AMD	80.67		11.25	6.23	4.58	2.28	☆☆☆☆☆☆☆
Kitagawa [26]	2016	16	4	20	20	Japan	AMD & PCV	70	11	9.9	9.8	4.07	3.19	☆☆☆☆☆
Kumar [27]	2016	6	4	10	10	India	AMD	66.9	7.3	5	3.1	-	-	☆☆☆☆☆
Lee [28]	2016	14	11	25	25	Korea	AMD	67.6	8.9	7.2	8.2	7.5	5.0	☆☆☆☆☆
Rickmann [32]	2021	6	11	17	17	Germany	AMD	81.7	5.2	3.3	1.6	-	-	☆☆☆☆☆☆☆
Silva [25]	2016	4	4	8	8	UK	AMD	81	4.3	3	1	6.0	5.2	☆☆☆☆☆
Treumer [23]	2010	3	9	12	12	Germany	AMD	81.5	5.4	4.8	3.8	4.3	3.2	☆☆☆☆☆

Abbreviations: No., number; SD, standard deviation; DD, disc diameter; NOS, Newcastle–Ottawa Scale; G1, Group 1; G2, Group 2; G3, Group 3; AMD, age-related macular degeneration. Note: “☆” represents the score of the methodological quality of the included studies according to the modified version (nine-star scoring system) of the NOS.

**Table 2:** Treatment regimens received by the included study participants.

Author	Publication Time (Year)	Operations						Complication Rate	Displacement Rate		Postoperative Position	
		PPV	SRI TPA	IVI TPA	SRI Anti-VEGF	IVI Anti-VEGF	Gas Tamponade		Total	Subtotal	Position	Time (Days)
Arias [22]	2010	+	+	+		+	+	20%	100%			
Avcı [20]	2021	+	+		+		+		53.3%	46.7%	Reading position	5
Boiche [29]	2019	+	+			+	+	15.4%	81%		Head down	3
Erdogan [30]	2020	+	+			+					Lying down	1–3
Grohmann (G1) [31]	2020			+		+	+	2.4%	100%		Prone position	At least 7
Grohmann (G2) [31]	2020	+	+			+	+					
Grohmann (G3) [31]	2020	+		+		+	+					
Guthoff [24]	2011			+		+	+				Prone position	2
Kitagawa [26]	2016			+		+		20.0%	85%	15%	Prone position	2
Kumar [27]	2016	+	+		+		+	20.0%	100%			
Lee [28]	2016			+		+	+	8.0%			Prone position	At least 7
Rickmann [32]	2021	+	+			+	+	17.6%	47%		Face down	
Silva [25]	2016			+		+	+	37.5%	100%		Face down	5
Treumer [23]	2010	+	+		+		+	33.3%	75%	25%	Prone position	1

Abbreviations: PPV, pars plana vitrectomy; SRI, subretinal injection; TPA, tissue plasminogen activator; IVI, intravitreal injection; anti-VEGF, anti-vascular endothelial growth factor.

**Table 3:** Follow-up of vision (logMAR).

Author	Publication Time (Year)	Preoperative BCVA		Postoperative BCVA										Final BCVA	
				1 Month		2 Months		3 Months		6 Months		12 Months			
		Mean	SD	Mean	SD	Mean	SD	Mean	SD	Mean	SD	Mean	SD	Mean	SD
Arias [22]	2010	1.5	0.2											1.1	0.3
Avcı [20]	2021	2.11	0.84	1.32	0.91	0.94	0.66	1.13	0.84	1.0	0.7			1.0	0.7
Boiche [29]	2019	2.4	0.6	1.8	1.0					1.6	1.0			1.7	1.0
Erdogan [30]	2020	2.46	0.64											1.73	0.86
Grohmann (G1) [31]	2020	1.41	0.48							1.05	0.52			1.05	0.52
Grohmann (G2) [31]	2020	1.46	0.54							1.28	0.61			1.28	0.61
Grohmann (G3) [31]	2020	1.63	0.53							1.33	0.59			1.33	0.59
Guthoff [24]	2011	0.92	0.89	0.6	0.59					0.62	0.46			0.62	0.46
Kitagawa [26]	2016	0.66	0.4							0.42	0.45			0.42	0.45
Kumar [27]	2016	1.45	0.3	0.95	0.4			0.86	0.5	0.82	0.6			0.82	0.6
Lee [28]	2016	1.09	0.77	0.78	0.61			0.6	0.54	0.54	0.56	0.52	0.6	0.52	0.6
Rickmann [32]	2021	1.37	0.39					1.03	0.57					1.03	0.57
Silva [25]	2016	1.67	0.47											0.63	0.33
Treumer [23]	2010	1.87	0.89	1.17	0.7			0.91	0.5					0.91	0.5

Abbreviations: BCVA, best-corrected visual acuity; SD, standard deviation; logMAR, logarithm of the minimum angle of resolution. Note: Final BCVA is the final visual acuity at the last time point during follow-up or the final BCVA explicitly reported in the literature.

**Table 4:** Follow-up of foveal thickness.

Author	Publication Time (Year)	Preoperative Foveal Thickness (μm)		Postoperative Foveal Thickness (μm)						Final Foveal Thickness (μm)	
				1 Month		3 Months		6 Months			
		Mean	SD	Mean	SD	Mean	SD	Mean	SD	Mean	SD
Grohmann (G1) [31]	2020	764	340					246	153	246	153
Grohmann (G2) [31]	2020	987	441					294	166	294	166
Grohmann (G3) [31]	2020	642	322					418	364	418	364
Kitagawa [26]	2016	599	319					208	71	208	71
Lee [28]	2016	562	183	244	85	215	58	250	119	266	107
Rickmann [32]	2021	607	179			423	205			423	205
Silva [25]	2016	658.1	174.2							316.6	142.4

Abbreviation: SD, standard deviation. Note: Final foveal thickness is the foveal thickness at the last time point during follow-up or the final foveal thickness explicitly reported in the literature.

## Data Availability

The data that support the findings of this study are available from the online database.
